# European Public Health News

**DOI:** 10.1093/eurpub/ckac025

**Published:** 2022-04-01

**Authors:** Dineke Zeegers Paget, Dineke Zeegers Paget, Elena Petelos, Catharina de Kat, Siddhartha Sankar Datta, Nino Berdzuli, Hans Henri P Kluge, Reinhard Busse, Floris Barnhoorn

**Affiliations:** 1 EUPHA Executive director; 1 EUPHA Executive Director; 2 Chair of the EUPHA working group on gender equality; 1 WHO Regional Office for Europe; 1 Chair 15th EPH Conference 2022; 2 EPH Conference Office

In this European public health news, we are touching upon urgent public health issues where European and even global collaboration is essential. De Kat et al. present the European Immunization agenda 2030, endorsed by all 53 countries in the European Region. This agenda intends to keep immunization as a priority on the political agenda and—as vaccination is a joint responsibility—on the agenda of all European citizens. Zeegers and Petelos address the continued problem of sexual violence in Europe and beyond and put down some pointers to change our way of looking at and dealing with sexual violence. This also requires political will and citizens’ awareness. Busse updates us on the upcoming European public health conference, where all these issues are presented under the main theme of building back better (and fairer) health systems.

## #MeToo, denial and the way forward—a global and public health priority

Zeegers PagetDineke^1^PetelosElena^2^ 1
EUPHA Executive Director
2
Chair of the EUPHA working group on gender equality

In 2006, Tarana Burke used the #MeToo for the first time to advocate women who survived sexual violence or harassment to openly share their experiences to fight sexual misconduct. In 2017, the hashtag went viral, bringing sexual abuse, harassment and violence to political agendas across the globe (MeToo movement—Wikipedia, last accessed 21 February 2022).

The United Nations Declaration on the elimination of violence against women defines violence against women as ‘any act of gender-based violence that results in, or is likely to result in, physical, sexual or psychological harm or suffering to women, including threats of such acts, coercion or arbitrary deprivation of liberty, whether occurring in public or in private life’ (The United Nations Declaration on the Elimination of Violence against Women, December 1993). By the time the #MeToo movement exploded, and despite the UN commitment, there was little progress in terms of concrete measures and of cultural and behavioural patterns radically shifting.

So how big is this problem? In the October 2021 factsheet, WHO reported that nearly one in three women worldwide have experienced physical and/or sexual violence by an intimate partner or sexual violence by any perpetrator in their lifetime (WHO-RHR-19.16-eng.pdf, last accessed 21 February 2022). In Europe, it is estimated that one in four women (this is 49 million women we are talking about) have or will experience physical and/or sexual violence by an intimate partner at some point in their life (WHO/Europe | Violence against women http://www.euro.who.int/en/health-topics/disease-prevention/vi…, last accessed 21 February 2022).

The #MeToo movement transformed perceptions and expectations: sexual harassment would no longer be condoned or tolerated, it would not be perceived and remain unaddressed as a ‘normal, expected, inevitable or unavoidable’ phenomenon. People around the world are, now, being made aware of the size of this problem. Even in high-income and pro-feminist countries like the Netherlands or the UK, physical and psychological sexual violence against women is very present. There is ample evidence on the need to redress imbalance of power or hierarchies—illiteracy, unemployment, low socioeconomic status and access to societal goods are all contributing factors. Another critical aspect the #MeToo was a catalyst for, was removing the stigma that was inextricably linked with reporting such behaviours. When members of the government (UK) or the parliament (The Netherlands) are now regularly accused of overstepping moral boundaries, we need to do more than just raise awareness and empathy. We need to address the denial. Women in prominent roles, across the world, have started speaking up about past experiences and how they had been victimized by men abusing their power.

The denial is something that large companies and institutions, as well as men with power, often tried to use to decrease the size of the problem. When the football director of a certain Amsterdam club was accused of overstepping moral boundaries with female staff, he made his apologies stating that he was not aware that his actions were offensive to women. Which is a surprise given the fact that he was sending ‘dick-pics’ to these women. Another, even bigger recent scandal in The Netherlands was within the setting of a popular TV programme, where unwanted and inappropriate sexual harassment was performed by men in powerful positions and young talents being pushed into activities they did not agree on. That the producer of the show—trying to accept responsibility—declared that the women should come forward if we want to change things without addressing the responsibilities of the men, led to another outcry against male dominance and abuse of power. Interestingly, and rather expectedly, in countries lagging behind in terms of equality (EIGE Index 2021), the #MeToo has and is taken longer to emerge, and reporting remains largely confined to cases of extreme violence.

Shame and deniability are the reasons that over 95% of this kind of behaviour went unpunished. Fortunately, #MeToo has changed this and women now dare to come forward. The importance is to ensure that this topic stays on the political agenda and that the women coming forward are well supported, ensuring retaliation or stigmatization do not pose a concrete threat both in terms of ensuring they do not further suffer in terms of their wellbeing, as well as in terms of preventing others from being vocal about their experiences. Only then can real change be made.

So what needs to change? We need to:

- Clearly define what constitutes sexual violence and establish who is most vulnerable to suffer it (children, women, sexual or ethnic minority groups, those socioeconomically deprived);
- Admit that sexual violence is a worldwide problem in society, with relevance and impact to everyone’s life, and needs to be addressed as such;
- Redefine societal values and examine the implementation thereof ensuring robust data collection (disaggregated data);
- Stop the denial culture and make men take responsibility for their actions; stop treating men exhibiting such behaviour like adolescents; share values through education and ensure professional learning;
- Sexual education, as well as education about human rights and their implementation, should start from primary schools;
- Create real possibilities for women to come forward without the risk of repercussions and fear of retaliation, across settings, and in terms of what this means for the financial independence, employability, family and socioeconomic status in general;
- De-shame ‘if we wear nice clothes, we are not asking for it’—learn that everyone has the right to express oneself and dressing is a way of expression—not a method to attract a sexual partner.

The big question is—of course—what would YOU do when faced with psychological or physical sexual violence?

## Protecting health through the European Immunization Agenda 2030 is everybody’s business

de KatCatharina^1^Sankar DattaSiddhartha^1^BerdzuliNino^1^KlugeHans Henri P^1^ 1
WHO Regional Office for Europe

At this juncture, over 2 years into the COVID-19 pandemic, vaccination has become a priority topic in the news and everyday conversations—and rightfully so. Together with public health and social measures, available and approved COVID-19 vaccines have already prevented hundreds of thousands of deaths in the WHO European Region alone (https://www.euro.who.int/en/media-centre/sections/press-releases/2021/who-regional-office-for-europe-nearly-half-a-million-lives-saved-by-covid-19-vaccination-in-less-than-a-year), last accessed 1 March 2022.

While COVID-19 vaccination has naturally taken centre stage, many other vaccines continue to protect us all every day: immunization has made and will continue to make important contributions in all countries to reducing childhood morbidity and mortality, particularly from pneumonia, diarrhoea and measles, and to improving the health and well-being of individuals across the life course.

With our focus on COVID-19 vaccines, we may have missed some of the good news in other areas: no measles-related deaths have been reported to date for 2021 in the WHO European Region (WHO/Europe | Vaccines and immunization—WHO EpiData); polio was detected in November 2020 in Tajikistan and its spread quickly interrupted (WHO/Europe | Vaccines and immunization—No polio detected in Tajikistan since third round of supplemental immunization); and data from the UK published in 2021 revealed that HPV immunization has almost eliminated cervical cancer in women in the country born since September 1995. (The effects of the national HPV vaccination programme in England, UK, on cervical cancer and grade 3 cervical intraepithelial neoplasia incidence: a register-based observational study—*The Lancet*.) These contributions exemplify the value of vaccines as a highly effective public health intervention tool.

As the unprecedented effort to rollout vital COVID-19 vaccines continues, the pandemic has impacted the capacity of countries to sustain routine immunization coverage, leading to a limited but nevertheless concerning backsliding in the WHO European Region in 2020 (WHO/Europe | Vaccines and immunization—Varied impact of COVID-19 on routine immunization in the European Region). The task of ‘building forward stronger’, based on the lessons learned from this pandemic includes ensuring that basic services, like routine immunization, are further strengthened—not only to make up for any lost ground but to make them fit for purpose in a new, very different decade.

Achieving and sustaining high and equitable vaccination coverage is fundamental to securing the greatest impact with vaccines and accomplishing disease-specific goals. For immunization systems in the WHO European Region, this means immediately putting in place catch-up services for all missed doses of routine vaccines in national immunization schedules, and in the longer term, working to ensure equitable access to vaccines regardless of age, location or population group; evidence-based outreach activities tailored to local needs; and sustainable financing for immunization.

The European Region’s 53 Member States took on this challenge to ‘build forward better’ in September 2021, by unanimously endorsing the European Immunization Agenda 2030 (EIA2030) (WHO/Europe | Vaccines and immunization—Countries of the WHO European Region adopt new regional immunization agenda for coming decade), a unique, clear vision and framework for action for the next decade, at the 71^st^ session of the WHO Regional Committee for Europe. It was formulated through a consultative process involving Member States and partners, and is one of the flagship initiatives of the European Programme of Work, 2020–2025—‘United Action for Better Health in Europe’ (EPW). For this flagship to achieve its stated aims, its joint development and unanimous adoption must be followed by inclusive implementation. A policy document is only words on a page unless it is used to guide, inspire and call for action.

### Departure from the past—unique elements of EIA2030

EIA2030 departs from earlier strategies by creating mechanisms for inclusion and accountability at all levels. Guided by the principle of ‘leaving no one behind’ enshrined in the EPW and the 2030 Sustainable Development Agenda, implementation of EIA2030 means prioritizing immunization equity. The ultimate goal is to protect all people from preventable suffering, and the way to achieve this is through stronger immunization systems at local and national levels.

By endorsing EIA2030, Member States have committed to:

- increase local ownership to solidify awareness of the importance of immunization and devise evidence-based local solutions to local challenges;
- harness political momentum to ensure that appropriate resources are allocated;
- strengthen the life-course vaccination platform to address the needs of adult populations;,
- mainstream catch-up vaccination;
- implement clearly defined results-based monitoring and accountability mechanisms at regional, national and subnational levels, with a robust monitoring and evaluation (M&E) and accountability framework.

### Shared roles and responsibilities for the benefit of all

As COVID-19 has brought into sharp focus, vaccination is a joint responsibility.

Keeping immunization as a priority on the political agenda will require steady and comprehensive engagement. This year, the WHO Regional Office for Europe will establish an immunization board composed of a broad range of stakeholders including Her Royal Highness The Crown Princess of Denmark (Patron of the WHO Regional Office), to keep the implementation of EIA 2030 on track.

WHO and its Member States have also agreed to hold each other accountable for their roles in making the ambitious targets a reality using a robust monitoring and evaluation (M&E) and accountability framework.

Now this commitment and joint agenda will have to be translated into evidence-based and funded national and subnational action plans that inspire and engage all stakeholders. Every vaccinator in every clinic should feel supported and inspired by the ambitions of EIA2030 that they are helping to achieve.

Individual actions matter—starting at home, everyone should check that they and their families are up to date on routine vaccinations and advocate to their local leadership for better access to health services where they are lacking. Misinformation about COVID-19 vaccines has amplified concerns and increased hesitancy in many countries. Increased politicization around this topic will impact perceptions and trust of other routine immunization programmes in the years to come. Seeking information on any vaccine-related concerns should be viewed as a ‘health-seeking behaviour’ and an opportunity to engage with the public—inclusiveness must be embedded in EIA2030 implementation.

There is not one day to waste. Every gap in immunization coverage is a window of opportunity for a preventable disease to strike.

24–30 April 2022 is European Immunization Week—an annual event to raise awareness of the importance of immunization in preventing diseases and protecting life, and we invite you to take part. Your contributions will help save lives by spreading the word that #VaccinesWork. Immunization is everyone’s business—and the EIA2030 is our opportunity to protect this generation and the next.

## 15th European Public Health Conference, 9–12 November 2022, Berlin, Germany

BusseReinhard^1^BarnhoornFloris^2^ 1
Chair 15th EPH Conference 2022
2
EPH Conference Office

Strengthening health systems: improving population health and being prepared for the unexpected

### Welcome to Berlin 2022

The 15th EPH Conference in Berlin will be the first EPH Conference since Marseille 2019 to take place live and in person from 9 to 12 November. The spectacular hub27 Berlin venue provides us with ample opportunities for informal chatting, personal exchange and live networking. We hope you will join us and look forward to welcoming you in Berlin.

### Themes for Berlin 2022

The theme of Berlin 2022 is *‘Strengthening health systems: improving population health and being prepared for the unexpected’*. In the past years, we have learned from the COVID-19 pandemic that infectious diseases know no boundaries. Although we have seen the successful development of vaccines, we have also seen with the spread of the Delta and Omicron variants around the globe that these vaccines have not yet been available to everyone. At the conference, we want to explore ways in which the public health community can rally around the WHO health system framework to bring about better population health. The growing health inequalities around the globe leads to a simple conclusion: Europe must take on a more active role in global health. This includes taking a stand on expansive issues such as planetary health, climate change, environmental degradation, water and food security, migration, gender and poverty. Europe must use its knowledge and influence to balance out market forces, expand regulatory oversight and create socially responsible systems for health, especially when it comes to the distribution of vaccines and essential medicines.

Digitalization is a key area where recent developments require us to exchange ideas and come up with new approaches. The past decades have seen significant developments in data collection and processing capacity as well as eHealth applications. We need to ensure that the vast amounts of data generated are well protected on the one hand, but also being available and usable for public health interventions. We want the discussions at the 15th EPH Conference to contribute to resilient health systems, universal health coverage (UHC), a well-trained and highly qualified clinical and public health workforce, and governance structures that take a stand and shape health systems globally.

The tentative plenary programme looks as follows:

Achieving universal health coverage (UHC): reducing health inequity
Organized by WHO Regional Office for Europe
Advocacy, information and communication for better health
Organized by EUPHA
Strengthening health system performance
Organized by European Observatory on Health Systems and Policies and the European Commission
Incorporating behavioural and cultural insights in health promotion
Organized by EuroHealthNet
Sustaining high-quality care: interprofessional training for our clinical and public health workforce
Organized by ASPHER and EHMA

### Abstract submission for Berlin 2022

Abstract submission is open until 1 May 2022, 18:00 CET. Participate at Berlin 2022 by sharing your work with colleagues. The EPH Conference offers excellent opportunities to learn from the latest research and practice, to network with experts and colleagues and to expand your professional horizons. Abstracts are invited for workshops and single presentations in all areas of public health research, practice, policy and education. Abstracts are considered for workshops, oral, pitch and poster presentations. Do not miss the opportunity to share your work at the largest public health event in Europe. Detailed instructions can be found on our website.

### Abstract mentor programme

This year again, we offer in collaboration with EUPHAnxt an Abstract Mentor Programme. The programme is meant for young and/or less experienced abstract submitters to receive feedback from experienced reviewers. The programme is especially targeted at researchers who have limited access to colleagues to ask for guidance and comments on their proposed abstracts. The programme is independent of the abstract review and selection process. Experienced reviewers from the International Scientific Committee and EUPHA Sections will act as mentors. For information here.

### Registration for Berlin 2022

Registration opens 1 April 2022. Information on registration fees will be available by mid-March 2022.

